# RhoC Modulates Cell Junctions and Type I Interferon Response in Aggressive Breast Cancers

**DOI:** 10.3389/fonc.2021.712041

**Published:** 2021-08-26

**Authors:** Hannah G. Abraham, Peter J. Ulintz, Laura Goo, Joel A. Yates, Andrew C. Little, Liwei Bao, Zhifen Wu, Sofia D. Merajver

**Affiliations:** Department of Internal Medicine, University of Michigan, Ann Arbor, MI, United States

**Keywords:** RhoC, breast cancer, junctions, interferon, TNBC

## Abstract

Metastases are the leading cause of death in cancer patients. RhoC, a member of the Rho GTPase family, has been shown to facilitate metastasis of aggressive breast cancer cells by influencing motility, invasion, and chemokine secretion, but as yet there is no integrated model of the precise mechanism of how RhoC promotes metastasis. A common phenotypic characteristic of metastatic cells influenced by these mechanisms is dysregulation of cell-cell junctions. Thus, we set out to study how RhoA- and RhoC-GTPase influence the cell-cell junctions in aggressive breast cancers. We demonstrate that CRISPR-Cas9 knockout of RhoC in SUM 149 and MDA 231 breast cancer cells results in increased normalization of junctional integrity denoted by junction protein expression/colocalization. In functional assessments of junction stability, RhoC knockout cells have increased barrier integrity and increased cell-cell adhesion compared to wild-type cells. Whole exome RNA sequencing and targeted gene expression profiling demonstrate decreased expression of Type I interferon-stimulated genes in RhoC knockout cells compared to wild-type, and subsequent treatment with interferon-alpha resulted in significant increases in adhesion and decreases in invasiveness of wild-type cells and a dampened response to interferon-alpha stimulation with respect to adhesion and invasiveness in RhoC knockout cells. We delineate a key role of RhoC-GTPase in modulation of junctions and response to interferon, which supports inhibition of RhoC as a potential anti-invasion therapeutic strategy.

## Introduction

Cancer metastases are the leading cause of death in cancer patients, and yet details of the cellular processes that drive the early metastases in aggressive cancers are not fully understood. RhoC, a member of the Rho GTPase family, has been linked to the metastatic potential of a variety of cancers including inflammatory breast cancer, pancreatic cancer, and melanoma (1–5). In breast cancer, RhoC expression correlates with increasing breast cancer stage and grade (as a histologic surrogate for aggressiveness), and higher RhoC expression was associated with higher patient mortality (6). Moreover, in this historical cohort, high RhoC was a predictor of poor response to standard chemotherapy regimens, increasing the likelihood that patients would experience metastasis and relapse (6). RhoC is overexpressed in the majority of cases of inflammatory breast cancer, the most aggressive and metastatic form of breast cancer (7). Animal and *in vitro* studies demonstrated that RhoC is necessary specifically for facilitating metastasis, primarily through protecting metastatic cells from apoptosis, modulating cell motility, and influencing chemokine secretion (8, 9).

These studies led us to postulate that a possible cellular effect of RhoC-driven metastatic progression is through modulation of cell junctions that would signal to motility and evasion of apoptosis. The Rho family GTPases regulate actin cytoskeleton organization (10), and thereby interact directly or indirectly with components of adherens junctions (AJs) and tight junctions (TJs) (11). RhoA, whose amino acid sequence is 90% homologous to RhoC (12), specifically is important for both the initial formation and the structural maintenance of AJs and TJs (13). Indeed, studies using both dominant negative and constitutively active forms of RhoA result in AJ and TJ instability (13, 14), suggesting that the stability of epithelial junctions is dependent on balanced activation of Rho GTPases.

When considering pathological settings of junction instability, the metastatic process itself is a prime example. Multiple studies of diverse cancer types demonstrate a loss of junction markers in malignant *vs* normal tissue; however, these studies differ on the prognostic value derived from the loss or dysregulation of junctions (15, 16). In a study of colorectal cancers, decreased E-cadherin and ZO-1 expression in primary tumors predicted which tumors went on to have liver metastases (17). In addition to the observed dysregulation of cell-cell junctions in the metastatic process, junction proteins are also known to be downregulated in settings of increased inflammatory interferon signaling (18, 19) and treating cells with interferon-alpha (IFN-α) leads to increased RhoA activation (20, 21). Furthermore, breast cancer tumors with high interferon signaling pathway expression are nearly twice as likely to metastasize compared to tumors with low levels of expression (22).

This study aims to investigate the role of RhoC in regulating cell-cell junction stability and interferon signaling in aggressive breast cancer cell lines. We assess the hypothesis that RhoC amplifies interferon signaling and thereby increases junction dysregulation, consequently promoting cancer cells’ motility and invasiveness; this work supports inhibition of RhoC as a potential therapeutic strategy in aggressive cancers.

## Materials and Methods

### Cell Culture and Reagents

MDA-MB 231 (MDA 231) cells were acquired from ATCC and maintained in Gibco RPMI-1640 (+) L-glutamine, 10% fetal bovine serum (FBS), 5 μg/mL gentamycin, and 1X anti-anti. VARI068 cells, sourced from a patient-derived xenograft (23), were maintained the same way. SUM 149 cells and SUM 190 cells were provided by Dr. Steve Ethier and were maintained in Gibco Ham’s F12 (+) L-glutamine, 0.5% penicillin-streptomycin, 2.5 μg/mL Amphotericin B, 5 μg/mL gentamycin, 5 μg/mL insulin, and 1 μg/mL hydrocortisone. SUM 149 cells were additionally supplemented with 5% FBS, while SUM 190 cells were supplemented with 0.1% bovine serum albumin. MCF7 cells were acquired from ATCC and maintained in DMEM, 10% FBS, 5 μg/mL gentamycin, and 1X anti-anti. MCF10A cells were acquired from ATCC and maintained in 50:50 DMEM:F12, 5% horse serum, 10 μg/mL insulin, 0.02 μg/mL epidermal growth factor, 0.5 μg/mL hydrocortisone, 0.1 μg/mL cholera toxin, 5 μg/mL gentamycin, and 1X anti-anti. All cells were maintained at 5% CO_2_, except for SUM 149 and SUM 190 which were maintained at 10% CO_2_. Interferon-alpha 2a (IFN-α) was obtained from GenScript (# Z03003-1), reconstituted in ddH_2_O, and used to treat cells at either 100 IU/ml or 1000 IU/ml.

### Generation of CRISPR-Cas9 Knockout Cells

As described in Allen et al. (24), SUM 149, MDA 231, VARI068, MCF7, MCF10A, and SUM 190 cells were transfected with pSpCas9(BB)-2A-GFP (PX458), provided by Feng Zhang (Addgene plasmid # 48138), containing the sequence GCCCTGATAGTTTAGGTGAG targeting RhoA for RhoA knockout lines or the sequence AGGAAGACTATGATCGACTG targeting RhoC for the RhoC knockout lines. Transfection was accomplished using the Nucleofactor II system (Lonza). 48 hours post-transfection, single cells were sorted by GFP expression and seeded into 96-well plates, and clonal expansion was carried out. Genomic DNA was then harvested from clones and screened for RhoA or RhoC mutations *via* SURVEYOR reactions (IDT) with the primer pair Forward-GTTTTAGACCGTCTGCCATTTC and Reverse-AATCTCCACCTACCAGGTTCAA for RhoA and Forward-CTGTCTTTGCTTCATTCTCCCT and Reverse-CCAGAGCAGTCTTAGAAGCCAT for RhoC. Clones that screened positive were subsequently sequenced to characterize their RhoA or RhoC mutations and were also immunoblotted for RhoA and RhoC.

### Antibodies

The following primary antibodies were used: anti-E-cadherin rabbit polyclonal antibody (ThermoFisher #PA5-32178) at 1:500 dilution, anti-β-catenin mouse monoclonal antibody (Invitrogen #MA1-300) at 1:500 dilution, anti-ZO-1 mouse monoclonal antibody (Invitrogen #33-9100) at 1:150 dilution for immunofluorescent staining and 1:200 for Western Blot, anti-Occludin rabbit polyclonal antibody (Zymed #71-1500) at 1:300 dilution for immunofluorescent staining and Western Blot, anti-p-STAT1 rabbit monoclonal antibody (CST #9167) at 1:1000 dilution, anti-STAT1 rabbit monoclonal antibody (CST #9172) at 1:1000 dilution, anti-p-STAT2 rabbit antibody (CST #4441) at 1:500 dilution, anti-STAT2 rabbit antibody (CST #4594) at 1:500 dilution, anti-IRF9 rabbit monoclonal antibody (CST #76684) at 1:500 dilution, anti-IFI27 rabbit polyclonal antibody (ThermoFisher #PA5-68038) at 1:1000 dilution, anti-IFITM1 mouse monoclonal antibody (Proteintech #60074-1-IG) at 1:20,000 dilution, anti-MX1 rabbit polyclonal antibody (Proteintech #13750-1-AP) at 1:1000, anti-ISG15 rabbit polyclonal antibody (Proteintech #15981-1-AP) at 1:1000, and anti-actin antibody (Sigma #A3854) at 1:15,000 dilution. The following secondary antibodies were used: Alexa Fluor 488-conjugated anti-mouse secondary antibody (Molecular Probes) at 1:1000 for immunofluorescent staining, Alexa Fluor 647-conjugated anti-rabbit secondary antibody (Molecular Probes) at 1:1000 for immunofluorescent staining, HRP-conjugated anti-mouse secondary antibody (Santa Cruz) at 1:4000 for Western Blot, and HRP-conjugated anti-rabbit secondary antibody (CST) at 1:2500 for Western Blot.

### Immunofluorescent Staining

Cells were seeded on 4-well chamber slides and grown to a confluent monolayer. Slides were fixed in 4% paraformaldehyde for 10 minutes at room temperature, washed with 100 mM PBS-glycine for 10 minutes at room temperature, then permeabilized in 0.1% Triton X-100 in PBS for 10 minutes at 4°C. Samples were washed thrice with 100mM PBS-glycine, then incubated in blocking solution containing IF Buffer (0.2% Triton X-100, 0.05% Tween-20, 0.1% BSA, 7.7mM NaN_3_ in PBS) and 10% goat serum for 1.5 hr at room temperature. Subsequently, samples were incubated in a primary antibody solution overnight at 4°C. The samples were then washed four times in IF Buffer for 15 minutes each at room temperature, then incubated in a secondary antibody solution (all secondary antibodies used at 1:1000 dilution), followed by one wash with IF Buffer for 20 minutes and two washes with PBS for 10 minutes each, at room temperature. Slides were mounted in Prolonged Gold Antifade reagent with DAPI (4′,6- diamidino-2-phenylindole) for nuclear counterstaining (Molecular Probes). Images were acquired on a Nikon A1B confocal microscope at 40X magnification.

### Western Blot

Protein lysates were mixed with loading dye and boiled at 95°C, then loaded into a 4-15% polyacrylamide gel and run at 130-160 V for about 90 minutes. For blotting proteins smaller than 90 kDa, gel was subsequently removed from chamber and soaked in 20% methanol at RT for 5 minutes, then transferred to a PVDF membrane using the iBlot 2 Dry Blotting System. For proteins larger than 90 kDA, gel was removed from chamber and soaked in 20% methanol transfer buffer for 5 minutes, then transferred to a PVDF membrane using a BioRad Wet Transfer chamber running at 80 V for 75 minutes. After transfer, the membrane was blocked in 5% milk/TBST at RT for 1 hr, rinsed with TBST thrice for 5 minutes each, then incubated in primary antibody solution at 4°C overnight on shaker. The next day, the membrane was again rinsed with TBST, then incubated in secondary antibody solution (in 5% milk-TBST) at RT for 1 hr. Once again, the membrane was rinsed with TBST, and then incubated in developing reagent at RT for 2 minutes. Finally the membrane was placed in a chemilluminescence reader and the blot was recorded.

### FITC-Dextran Assay

Cells were seeded into Transwell plates and grown for 36 hours, until they reached confluency. FITC-Dextran solution was prepared at 1mg/ml, and 0.5ml of this solution was added to the apical chambers of the Transwells, with normal media in the basal chambers. After 24 hours, 50ul was removed from the basal chambers and transferred to a 96-well plate, then fluorescence was measured in a fluorescent plate reader. The ratio of fluorescence from the apical chamber to the basal chamber was recorded.

### Centrifugation Adhesion Assay

Adapted from Weetall et al (25). V-bottom 96-well plates were seeded with 2 x 10^4^ cells/well and left in 37°C overnight. Calcein-AM-labeled cells (2uM Calcein-DMSO solution, Invitrogen #C3100MP) were subsequently seeded at 1.5 x 10^4^ cells/well to the plate (negative control: wells with overnight-seeded cells but no Calcein-labeled cells; positive control: empty wells with Calcein-labeled cells added). Plates were incubated at 37°C for 2 hours, then centrifuged at 75 g for 10 minutes. Nonadherent cells accumulated at the bottom of the wells and fluorescence at the bottom of the well was quantified. Log fold change in fluorescence between test wells and positive control wells was recorded. Assay was repeated with media containing 100 IU/ml IFN-α; overnight-seeded cells were treated with IFN-α for 48 hours prior to seeding in v-bottom plates, then were seeded in media with IFN-α for 24 hours, while Calcein-labeled cells were treated with IFN-α for 72 hours prior to Calcein labeling, seeding, and incubation in v-bottom plate for 2 hours (they were also seeded in media containing IFN-α).

### siRNA Knockdown of Junction Proteins

siTJP1 (ZO-1) and non-targeting control siRNA were ordered from Dharmacon (siTJP1 5 nmol #L-0077-46-00-0005) and transfected in SUM 149s using 5.2 μl DharmaFECT 2/well in 6-well plates, while in MDA 231s transfection used 2 μl DharmaFECT 4/well in 6-well plates (Dharmacon). Protein was harvested from cells 2-5 days after transfection and immunoblotted for ZO-1 to confirm transient knockdown.

### Transwell Invasion Assay

100,000 cells/well were seeded into Matrigel Transwell Invasion chambers (Corning #354480) in serum-free media, with serum-containing media in bottom chambers. Cells were incubated at 37°C for 24 hours, then the top chambers were scrubbed to remove cells that had not invaded. Chambers were then fixed in 70% ethanol for 10 minutes, stained in 0.2% Crystal Violet for 10 minutes, and left to dry overnight. Brightfield images of each insert were acquired at 2X magnification on an Olympus IX51, and the ImageJ Color Inspector 3D plugin was used to quantify the percent coverage of purple pixels per insert image. Assays were performed in technical triplicate and biological triplicate. Multiple comparisons ANOVA was conducted on the data in GraphPad Prism 9. For assays with siRNA-treated cells, cells were seeded into chambers 48 hours post-transfection. For assays with IFN-α-treated cells, cells were seeded into chambers either with no prior IFN-α treatment or with 48 hours pre-treatment with IFN-α, and were seeded in serum-free media containing 100 IU/ml of IFN-α.

### RNAseq

In order to assess the impact of RhoC knockout on gene expression in breast cancers, a panel of cell lines was assembled: SUM 149 (triple-negative inflammatory breast cancer), MDA 231 (triple-negative non-inflammatory breast cancer), VARI068 (triple-negative non-inflammatory breast cancer), SUM 190 (hormone receptor negative, HER2 positive inflammatory breast cancer), MCF7 (estrogen receptor positive, progesterone receptor positive, HER2 negative non-inflammatory breast cancer), and MCF10A (normal-like breast epithelial cells). These cells all express varying levels of RhoC at baseline and vary in phenotype, from highly metastatic to noninvasive, and were chosen in order to assess whether RhoC knockout would induce gene expression changes that would be consistent across different cell contexts. Four biological replicates of all cell lines, wild-type and RhoC knockouts, were incubated at 37°C overnight. Normal growth media for each cell line was replaced with DMEM for 24 hrs, and RNA was harvested. RNA was sequenced *via* the Illumina HiSeq 4000 as paired 51bp reads to a targeted depth of 75M paired reads per sample. Read data in FASTQ format were quality assessed with FastQC/MultiQC (v.0.11.3) and contamination checked with fastq_screen (v.0.11.1). Reads were adapter-trimmed using CutAdapt (v.1.8.1) and aligned to the GRCh37 hg19 human genome using Tophat/Bowtie2 (v.2.0.13/v.2.2.1, options –b2_very_sensitive and the default max intron length of 500000). Raw read counts were extracted for each gene using HTSeq (v.0.6.0). DESeq2 (v1.14.1), run within the R (v.3.3.3) Bioconductor package (Biobase v.2.34) was used to model differential expression in genes between modeled conditions. The main factors used in the model were cell line and CRISPR knockout status (cRhoC or WT). DESeq2 utilizes generalized linear models for each gene and infers a log2 fold change between conditions using maximum likelihood estimation and (by default) a Wald test for significance. Default parameters for DESeq2 were used, specifying a standardized normal prior on the non-intercept coefficients (betaPrior=TRUE). QC plotting was performed in R using ggplot. Genes were annotated with NCBI Entrez GeneIDs and text descriptions. Data has been deposited in GEO, accession ID GSE175787.

In the crRhoC *vs* WT dataset comprising data from all cell lines listed above, 1293 differentially expressed genes were identified out of a total of 20,978 with detected expression based on an adjusted p-value threshold of 0.05 and a minimum absolute log2 fold change of 0.585. Gene set enrichment was performed on these data using the commercial iPathwayGuide software (Advaita Bioinformatics, Ann Arbor, MI). iPathwayGuide (iPG) scores pathways using a custom enrichment method (26–28) that is composed of two primary sub-methods: i) the over-representation of differentially expressed (DE) genes in a given pathway, and ii) the perturbation of that pathway computed by propagating the measured expression changes across the pathway topology. These two sub-methods each produce p-values (pORA and pAcc, respectively) that are combined using Fisher’s method into a pathway-specific p-value, which is then corrected for multiple comparisons using an FDR correction. The tool searched KEGG pathways (Release 90.0+/05-29, May 19) utilizing directional information in gene-relationships (29). An enrichment against GO terms (30, 31) was also performed, utilizing the ORA method (i) above.

In addition to classic enrichment, a prediction of upstream regulators was also performed by iPG based on the differentially expressed gene set and a network of regulatory (activation/inhibition) interactions from a proprietary knowledge base compiled from StringDB (32) (Version 11.0. Jan 19th, 2019) and BioGrid (33) (v3.5.171. March 25th, 2019) data. The activation/inhibition network is polled using gene expression information to consider hypotheses that upstream regulators of genes are either activated or inhibited. A z-score for each upstream regulator is computed by iterating over connected downstream genes and their incoming edges, as well as a p-value corresponding to the z-score as the one-tailed area under the probability density function for a normal distribution, N(0,1). An over-representation approach is also used to compute the statistical significance of observing at least a given number of consistent DE genes, with an associated p-value computed using the hypergeometric distribution (34). Finally, these two p-values are combined using the Fisher’s method to rank the upstream regulators and test the hypothesis that the upstream regulators are predicted as activated or inhibited in the experimental condition (crRhoC *vs* WT).

### Targeted Gene Expression Profiling

Three biological replicates of MDA 231 and SUM 149 wild-type, RhoA knockout, and RhoC knockout cells were treated with 100 IU/ml IFN-α for 72 hours, then RNA was harvested and run on nanoString Pan Cancer Immune Profiling panels (nanoString Technologies, Inc.). The expression of 730 immune-related genes and 40 housekeeping genes was measured, and the nSolver 4.0 software (nanoString Technologies, Inc.) was used to normalize expression values and conduct differential expression analysis. Genes were considered differentially expressed between treated and untreated cells if they had FDR-adjusted p-value < 0.05. To compare the change in expression with IFN-α treatment in wild-type, RhoA knockout, and RhoC knockout cells, p-values were calculated as per Kaye et al. (35).

## Results

### Loss of RhoA and RhoC Expression in Breast Cancer Cells Results in Significant Morphological Changes

**Figure 1:**
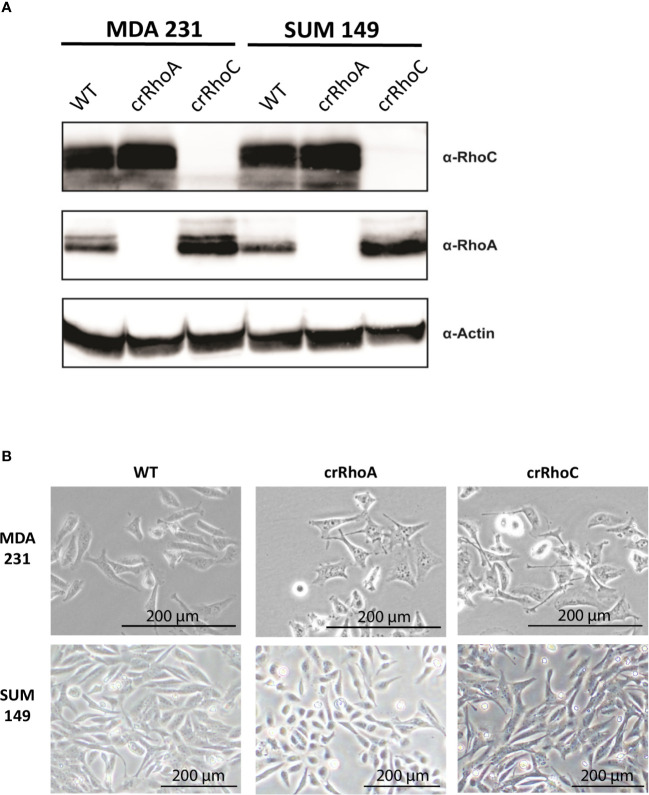
Rho knockout changes expression and morphology of cells. **(A)** Validation of CRISPR-Cas9 knockout of RhoA and RhoC *via* Western Blot. **(B)** Brightfield images of wild-type, crRhoA, and crRhoC cells. RhoA knockout markedly changes the morphology of both MDA 231 and SUM 149 cells, leading to a consistent “triangle” shape in the MDA 231s and a rounded shape in the SUM 149s. RhoC knockout changes cell morphology more subtly, leading to a consistent “crescent” shape in the MDA 231s and a more cuboidal shape in the SUM 149s. Scale bars = 200μm.

In order to investigate the effect of RhoC expression on cell-cell junctions, we created MDA 231 and SUM 149 cell lines where RhoA and RhoC had been independently knocked-out *via* CRISPR-Cas9 (cell lines denoted crRhoA and crRhoC, respectively). We found that the crRhoC cells exhibited compensatory increases in RhoA expression, while crRhoA cells had smaller magnitude increases in RhoC expression (**Figure 1A**). Furthermore, the crRhoA cells assumed a more spindlelike shape compared to their wild-type counterparts, and the crRhoC cells were more cuboidal compared to wild-type (**Figure 1B**).

### RhoA and RhoC Expression Modulate Junctional Protein Expression and Colocalization

**Figure 2:**
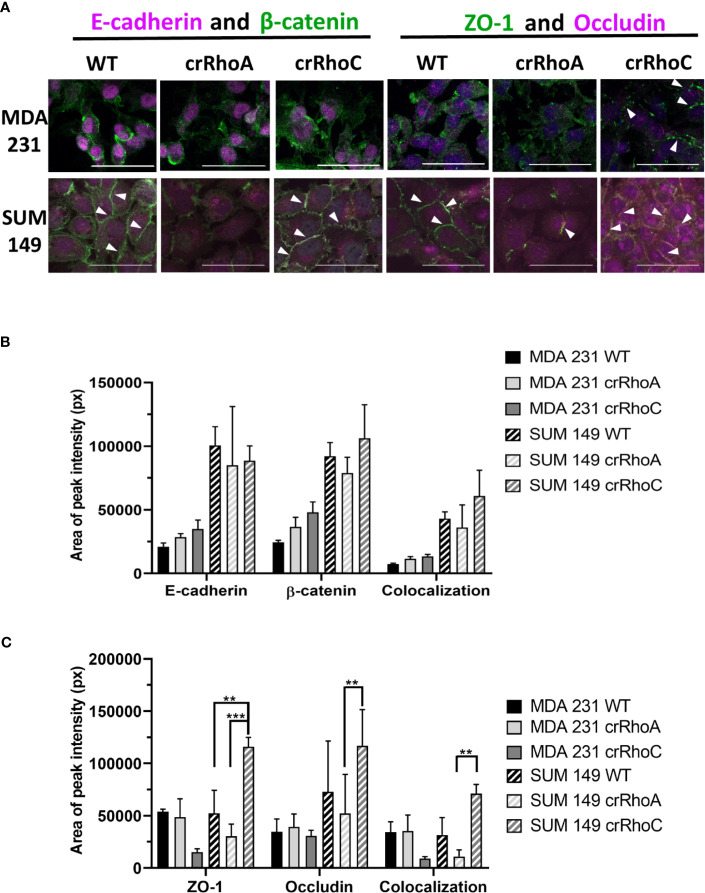
Rho expression changes junction marker localization to cell-cell borders. **(A)** Representative images of immunofluorescence staining of junction markers in wild-type, crRhoA, and crRhoC cells. White arrows point to areas of junction marker localization to cell-cell borders. Scale bars = 50 μm. Quantification from 3 fields of view per cell type of **(B)** adherens junction marker and **(C)** tight junction marker localization to cell-cell borders in wild-type, crRhoA and crRhoC cells, with area of peak intensity corresponding to areas of cell-cell border localization. Solid bars are MDA 231, striped bars are SUM 149; black bars are wild type, light grey bars are crRhoA cells, and dark grey bars are crRhoC cells. **p < 0.01; ***p < 0.001.

Due to the marked changes in morphology, we sought to characterize the role of RhoC and RhoA in epithelial junctions. We assessed the junctions structurally by the expression of the tight junction proteins ZO-1 and Occludin, and the adherens junction proteins E-cadherin and β-catenin. We found that crRhoA cells demonstrated similar or decreased expression of junction proteins as compared to wild-type *via* Western Blot. In contrast, crRhoC cells exhibited increased junction marker expression compared to wild-type (**Supplementary Figure 1**). Out of the four junction markers, this pattern of Rho-modulated expression was most evident in ZO-1, both in SUM 149 and in MDA 231 cells. Immunofluorescent staining for junction proteins (**Figure 2A**) highlighted an increase in the amount of junction markers localizing to areas of cell-cell contact in the crRhoC cells, as well as increased colocalization of junction markers in crRhoC cells. Moreover, the SUM 149 crRhoA cells were observed to consistently assemble in loose or disordered clusters, characterized by variable spaces between cells, and had decreased tight junctions and cell-cell projections compared to both wild-type and crRhoC cells (**Figures 2B, C**).

### RhoA and RhoC Expression Modulate Cell-Cell Adhesion and Barrier Impermeability

**Figure 3:**
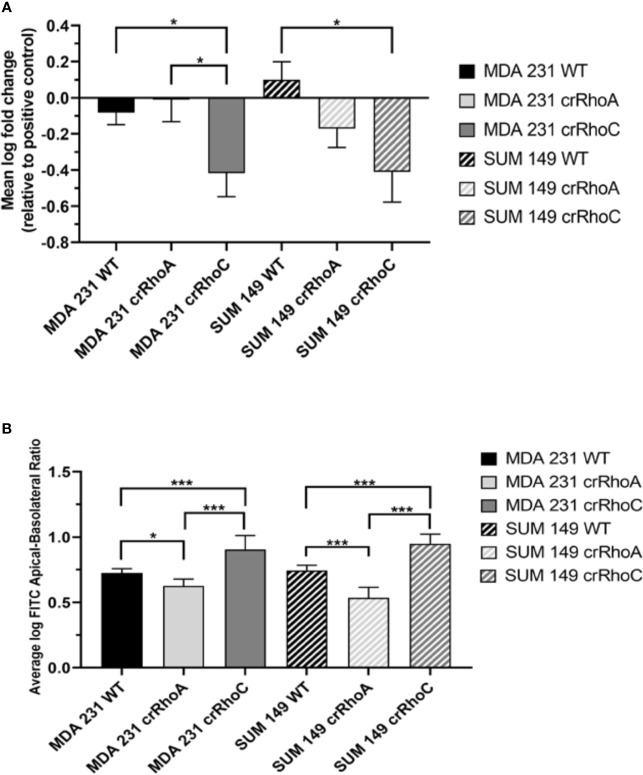
Rho expression changes cell-cell adhesiveness and junction stability. **(A)** Quantification of difference in fluorescent intensity between positive control (non-adhering) and test wells in centrifugation adhesion assay (n = 3 biological replicates); decreases in fluorescent intensity correspond to increases in cell-cell adhesiveness. crRhoC cells have significantly increased adhesiveness compared to wild type. **(B)** Quantification of the ratio of fluorescent intensity in apical *vs* basal chambers in FITC-Dextran barrier permeability assay (n = 3 biological replicates); higher ratio corresponds to increased barrier integrity. crRhoA cells have decreased barrier integrity compared to wild-type, whereas crRhoC cells have increased barrier integrity. Solid bars are MDA 231, striped bars are SUM 149; black bars are wild-type, light grey bars are crRhoA, and dark grey bars are crRhoC cells. *p < 0.05; ***p < 0.001.

Having observed a qualitative change in junction protein expression and localization, we sought to determine whether this change translated into functional differences in adhesion between wild-type and crRhoC cells. We measured cell-cell adhesion using a fluorometric centrifugation assay, wherein fluorescently-labeled cells were added to wells with previously-seeded cells of the same type, incubated for 2 hours, and then subjected to centrifugal shear stress in order to measure the perturbation of adhesion between different cell types. Both crRhoC SUM 149 and crRhoC MDA 231 cells had a greater reduction in fluorescent signal compared to their positive controls than did wild-type cells, suggesting a functionally stronger cell-cell adhesion when RhoC is knocked out (**Figure 3A**). To further assess the functional significance of the junction changes induced by reducing RhoA and RhoC expression, we undertook a FITC-Dextran barrier integrity assay to determine the effectiveness of the tight junctions in these cells. In both crRhoC SUM 149 and crRhoC MDA 231 cells, there was a significant increase in the barrier integrity of the cell monolayer compared to wild-type, and in crRhoA SUM 149 and crRhoA MDA 231 there was a significant decrease in the barrier integrity of the cell monolayer compared to wild-type (**Figure 3B**). These changes imply that tight junction stability increases with RhoC knockout, and decreases with RhoA knockout, which is consistent with the changes observed *via* immunofluorescent staining.

To investigate whether RhoA and RhoC expression affect the invasive potential of breast cancer cells, we conducted transwell invasion assays. Compared to wild-type and crRhoA cells, crRhoC cells had significantly less transwell invasion in MDA 231 cells and trended to less invasion in SUM 149 cells (**Supplementary Figure 2**). To assess whether transiently modulating expression of ZO-1 would contribute to invasive capability, cells were treated with ZO-1 siRNA or scrambled control siRNA for 72 hrs to achieve transient ZO-1 knockdown, following which transwell invasion was assessed. ZO-1 knockdown did not significantly change invasiveness in wild-type, crRhoA, or crRhoC cells (data not shown).

### crRhoC Cells Have Altered Interferon-α Signaling Compared to Wild-Type

**Figure 4:**
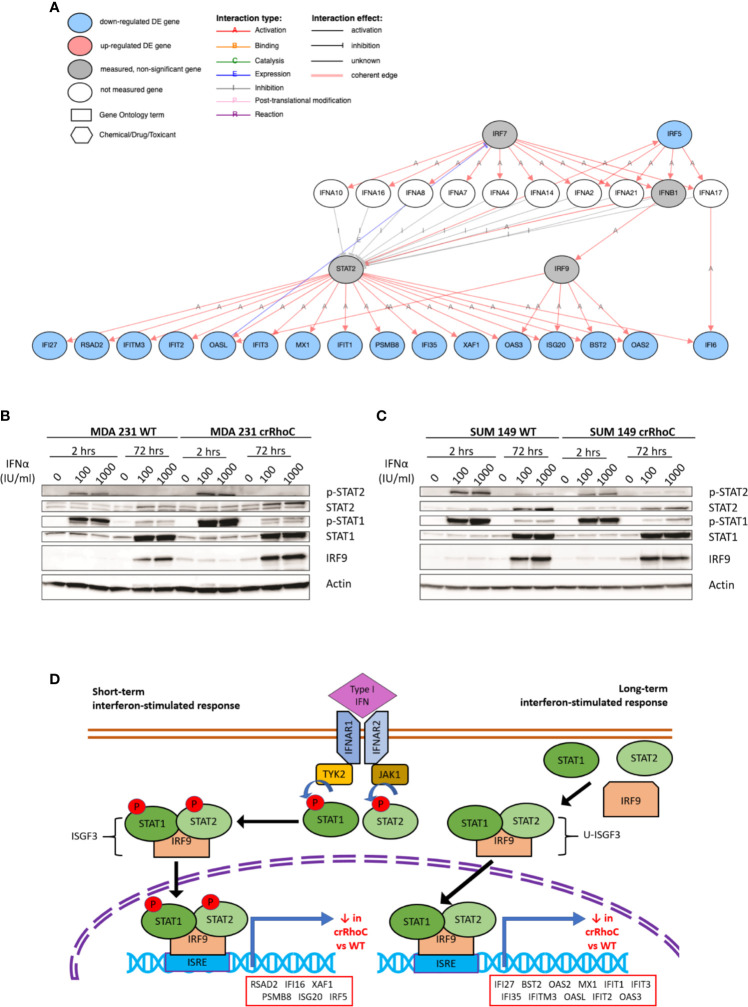
RhoC expression influences gene and protein expression of Type I interferon signaling response. **(A)** Type I interferon signaling pathway and the genes significantly downregulated in crRhoC knockout cells compared to wild-type, as measured in RNAseq. Genes in blue circles were downregulated in crRhoC compared to wild-type; genes in grey circles were not significantly differentially expressed between crRhoC and wild-type. **(B)** Western blot of interferon signaling markers in MDA 231 wild-type and crRhoC cells—crRhoC cells have increased p-STAT2 and IRF9 compared to wild type. **(C)** Western blot of interferon signaling markers in SUM 149 wild-type and crRhoC cells—crRhoC cells have decreased p-STAT2 and STAT2 compared to wild type. **(D)** Short-term and long-term signaling through type I interferon signaling pathways. Short-term interferon signaling is driven by phosphorylated STAT1 and STAT2 complexed with IRF9 that translocates to the nucleus, binds to interferon stimulated response elements (ISREs), and promotes transcription of interferon stimulated genes (ISGs); phosphorylation of STAT1 and STAT2 peak about 2 hours after treatment with a type I interferon. Long-term signaling is driven by unphosphorylated STAT1 and STAT2 complexed with IRF9, and peaks around 72 hours after treatment with type I interferons. Genes listed in order of decreasing magnitude of log fold-change.

Seeking to understand the molecular mechanisms of Rho-driven junction regulation, we conducted whole transcriptome RNAseq analysis of SUM 149, MDA 231, VARI068, MCF7, MCF10A, and SUM 190 wild-type and crRhoC cells. Analysis detected 1,293 genes differentially expressed between crRhoC cells and wild-type at an adjusted p-value of 0.05 and a minimum log2 fold change threshold of 0.585. A number of interferon-stimulated genes (ISGs) had significantly decreased expression in crRhoC cells compared to wild-type. Inferring the upstream regulation of genes from the overall differential expression result set yielded IRF9 and STAT2 as the two leading inhibitory regulators (**Supplementary Table 1**), with 17 consistent genes each listed as inhibited (out of a total of 38 and 43 target genes, p-values 7.9e-11 and 3.9e-10, respectively) (**Figure 4A** and **Supplementary Table 2**). The expression of IRF9 and STAT2 themselves were not significantly differentially altered between crRhoC cells and wild-type cells.

As type I interferon signaling is known to influence junction protein expression in a context-dependent manner (18, 36), we sought to investigate specifically whether the predicted inhibition of type I interferon signaling in crRhoC cells was borne out at the protein level, and whether any changes in junctional behavior would result. SUM 149 and MDA 231 wild-type and crRhoC cells were subsequently treated with IFN-α at two doses (100 and 1000 IU/ml) for 2 hours and 72 hours, and expression of proteins in the type I interferon signaling pathway was assessed *via* Western Blot. In response to interferon treatment, we observed that RhoC modified the cells’ responses: MDA 231 crRhoC cells had increased p-STAT2 and IRF9 expression compared to wild type (**Figure 4B** **)**, whereas SUM 149 crRhoC cells had decreased p-STAT2 and total STAT2 expression compared to wild type (**Figure 4C**). There were no significant differences between the two doses tested. The difference in interferon response expression between crRhoC and wild-type cells were evident at both the 2 hour and 72 hour time points, consistent with the 17 ISGs identified by RNAseq that are downstream of short-term phosphorylated STAT1-STAT2-IRF9-complex(ISGF3)-driven signaling as well as long-term unphosphorylated-ISGF3-driven signaling (37–39) (**Figure 4D**).

### RhoC Modulation of Interferon Signaling Leads to Functional Changes in Junction Behavior and Cell Invasiveness

**Figure 5:**
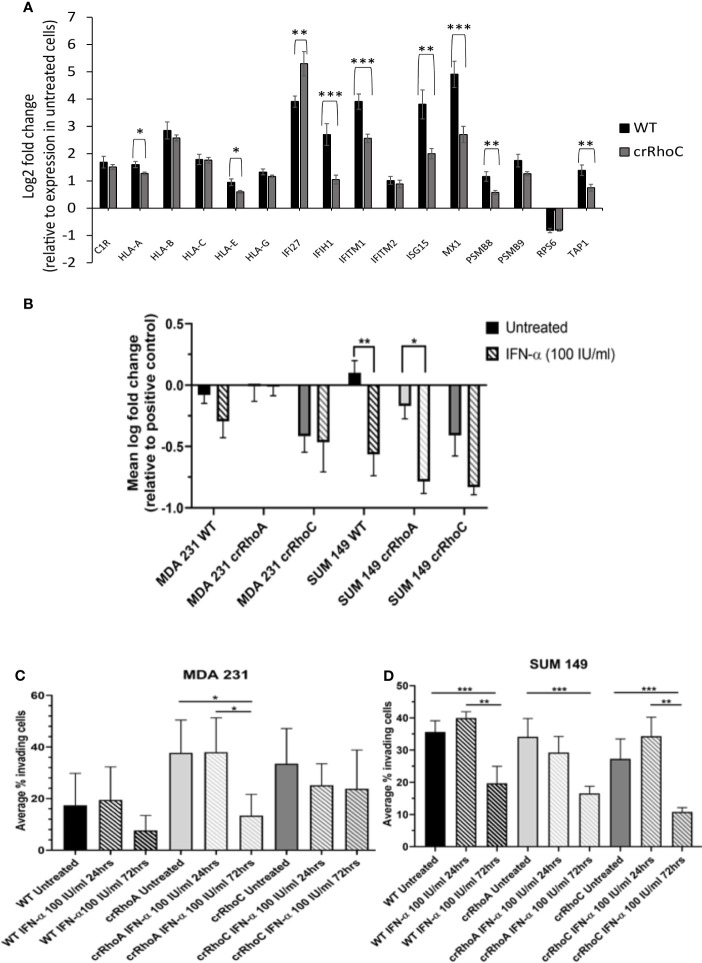
RhoC expression modulates cells’ functional response to interferon. **(A)** RNA expression of interferon stimulated genes in response to 72 hours of IFN-α treatment (100 IU/ml) in MDA 231 and SUM 149 cells. Wild-type cells had larger increases in gene expression with IFN-α treatment compared to crRhoC cells. Black bars are expression in wild-type cells, dark grey bars are expression in crRhoC cells. **(B)** Quantification of difference in fluorescent intensity with IFN-α treatment between positive control (non-adhering) and test wells in centrifugation adhesion assay (n = 3 biological replicates); decreases in fluorescent intensity correspond to increases in cell-cell adhesiveness. Treatment with IFN-α at 100 IU/ml for 72 hours led to increased adhesion for all cells, but the increases were larger and more significant in wild-type and crRhoA cells compared to crRhoC cells. **(C)** Quantification of transwell invasion with IFN-α treatment (n = 3 biological replicates) in MDA 231 and in **(D)** SUM 149. Treatment with IFN-α led to decreased invasion for all cells; in MDA 231 wild-type and crRhoA cells had larger and more significant decreases than in crRhoC cells, whereas in SUM 149 crRhoC cells had the largest relative decrease in invasion. Downward-slanting stripes represent IFN-α treatment; black bars are wild-type, light grey bars are crRhoA cells, and dark grey bars are crRhoC cells. *p < 0.05; **p < 0.01; ***p < 0.001.

To assess the impact of RhoA and RhoC expression on long-term ISG expression, SUM 149 and MDA 231 wild-type, crRhoA and crRhoC cells were treated with IFN-α at 100 IU/ml for 72 hours, then RNA was harvested and a relevant array of cancer related genes was assessed by the nanoString Pan-Cancer Immune Panel. Out of the genes that were significantly differentially expressed in treated cells compared to untreated controls, the interferon-stimulated gene IFITM1 had decreased expression in both crRhoA and crRhoC cells compared to wild type, and additional interferon-stimulated genes like MX1 and ISG15 were significantly decreased in only the crRhoC cells compared to wild type. IFI27 was the only interferon-stimulated gene that had significantly increased expression in treated crRhoC cells compared to both treated crRhoA cells and treated wild-type (**Figure 5A** and **Supplementary Figure 3A**).

In order to determine whether these differences in mRNA expression between crRhoC cells and wild-type cells were borne out at the protein level, cells were again treated with IFN-α at 100 IU/ml for 72 hours, and protein was harvested for Western Blot of MX1, ISG15, IFITM1 and IFI27 (**Supplementary Figure 3B**). There was no expression of these proteins in the untreated SUM 149 cells, whereas in MDA 231 the untreated cells all expressed ISG15 and the untreated crRhoA and crRhoC cells expressed IFI27, with crRhoC cells expressing the highest amount of these two proteins at baseline. The treated cells all had similar protein expression of MX1, ISG15, and IFI27, with MDA 231 cells having slightly increased protein expression compared to SUM 149 cells. However IFN-α treatment elicited higher IFITM1 protein expression in crRhoC cells than in wild-type. SUM 149 crRhoA cells had decreased expression of IFITM1 with IFN-α treatment compared to wild-type, whereas MDA 231 crRhoA cells had increased expression of IFITM1 with IFN-α treatment compared to wild-type.

We further sought to assess the impact of RhoA and RhoC expression on functional responses to IFN-α treatment. The centrifugation adhesion assay was repeated with media containing IFN-α at 100 IU/ml. 72 hours of IFN-α treatment increased cell-cell adhesion for all cell types. However, the change in adhesion between untreated and IFN-α treated cells was greater in magnitude in wild-type cells compared to crRhoC cells **(** **Figure 5B** and **Table 1** **)**. Transwell invasion assays were also repeated with media containing IFN-α at 100 IU/ml, with cells treated for 24 or 72 hours. Cells treated for 72 hours had reduced invasion compared to untreated cells and 24-hour-treated cells. In MDA 231, the magnitude of invasion reduction was greater in wild-type and crRhoA cells compared to crRhoC cells, whereas in SUM 149s the reverse was demonstrated—crRhoC cells had a larger reduction in invasion than wild-type or crRhoA cells (**Figures 5C, D** and **Table 2**). There were no significant differences in proliferation or viability between treated and untreated or between wild-type and Rho knockout cells (data not shown).

**Table 1:** RhoC knockout dampens IFN-α-driven increase in cell-cell adhesion.

Cell Line	|Log fold change| with IFN-α tx	p value with IFN-α tx
MDA 231 WT	3.63	0.250
MDA 231 crRhoA	1.30	0.982
MDA 231 crRhoC	1.12	0.864
SUM 149 WT	5.69*	0.037
SUM 149 crRhoA	4.59*	0.007
SUM 149 crRhoC	2.02	0.120

Adhesion was measured using the fluorimetric centrifugation adhesion assay. Log fold change in fluorescence corresponds to change in adhesiveness. Significance determined by p < 0.05 and indicated by *.

**Table 2:** RhoC knockout modulates IFN-α-driven inhibition of cell invasion.

Cell Line	Fold change with IFN-α tx 72 hr	p value with IFN-α tx 72 hr
MDA 231 WT	0.559	0.431
MDA 231 crRhoA	0.645*	0.012
MDA 231 crRhoC	0.289	0.809
SUM 149 WT	0.447*	2E-4
SUM 149 crRhoA	0.514*	1E-4
SUM 149 crRhoC	0.604*	9E-4

Cells were treated with IFN-α for 72 hours and transwell invasion assays were conducted. Fold change between the percent invading cells for untreated and treated cells was calculated. Significance determined by p < 0.05 and indicated by *.

## Discussion

In investigating the cellular and molecular basis of the impact of RhoC on metastasis, we demonstrate that RhoC affects both cell-cell junction behavior as well as IFN-α response. Knocking out RhoC results in a trend towards increased tight and adherens junction protein expression (**Supplementary Figure 1**) and membrane localization (**Figure 2**) that resembles normal junctions, while also significantly increasing the functionality of these junctions with respect to adhesiveness and impermeability (**Figure 3**). crRhoC cells also have decreased cell invasion (**Supplementary Figure 2**). Interestingly, low-dose IFN-α treatment has similar effects on wild-type cells as the effect of RhoC knockout—the increased adhesion and decreased invasion induced by 72 hours of IFN-α treatment in wild-type cells (**Figures 5C, D** **)** is comparable in magnitude to the increased adhesion and decreased invasion seen in crRhoC cells compared to wild-type. crRhoC cells treated with IFN-α exhibit dampened response in terms of changes in adhesion and invasion, compared to treated wild-type cells, but it is important to highlight that IFN-α does increase adhesion and decrease invasion in both crRhoC and wild-type cells. Taken together, these data point to IFN-α and RhoC inhibition as being capable of reducing cancer cell invasion in a cumulative fashion—a potential combination strategy that could be more effective in RhoC-driven phenotypes, such as inflammatory breast cancer, as there was clearly a larger effect on adhesion and invasion in SUM 149 crRhoC cells treated with IFN-α compared to MDA 231 crRhoC cells.

A corollary interpretation of these results is that RhoC knockout blunts cellular response to IFN-α overall. This interpretation is further supported by the smaller increase in expression of interferon-stimulated genes in crRhoC cells post-IFN-α treatment compared to wild-type cells, in which IFN-α treatment robustly increased interferon-stimulated gene expression (**Figure 5A**). The potential for RhoC contributing to normal IFN-α signaling is a novel finding. Expression of interferon signaling proteins IFI27 and ISG15 was higher in untreated MDA 231 crRhoC cells than in wild-type, and IFITM1 expression was higher in treated crRhoC cells than in wild-type. Expression of IFI27 in some studies is correlated with decreased proliferation and migration (40, 41), and in others with increased tumorigenesis and migration and decreased patient survival (42–44). ISG15 expression is correlated with increased invasion, induction of M2-like macrophages, and decreased patient survival (45, 46). IFITM1 is also correlated with increased tumorigenesis and invasion (47). The increased expression of these invasion-associated proteins and the overall decreased ISG expression in crRhoC cells compared to wild-type, in the context of IFN-α treatment reducing cell invasion without significantly affecting cell viability, adds complexity to the understanding of RhoC as primarily a promoter of metastasis.

Previous studies from our lab have found that macrophage-conditioned media, specifically from M2a macrophages, promotes cancer cell invasion, and that functional RhoC is necessary to achieve the full extent of macrophage-promoted invasion (24, 48). Interestingly, IFN-α treatment has been demonstrated to promote a shift in macrophage polarization from M2 to M1 (49, 50). Our current study posits that functional RhoC contributes to increased IFN signaling in cancer cells, which would conflict with the logical conclusion from previous studies that RhoC is positively associated with M2 macrophages and M2 macrophages are negatively associated with IFN-α. Further study is therefore necessary to determine why cells with functional high RhoC expression have reduced junction functionality and increased invasion in the absence of IFN-α, and the opposite effect in the presence of IFN-α.

IFN-α has been recognized as an anti-tumor compound since 1970 (51). High-dose IFN-α (>1000 IU/ml) is FDA-approved as monotherapy for Kaposi’s sarcoma, follicular non-Hodgkin lymphoma, melanoma, and hairy-cell leukemia, and for adjuvant therapy of melanoma; overall clinical response rates are modest, and high-dose IFN-α toxicity is high, thus oncological use has diminished in recent times (52). On the other hand, IFN-α is also used clinically as an anti-viral agent, and achieves sustained anti-virologic responses for significant populations of Hepatitis B and C patients (53). Some of the variation in clinical efficacy of IFN-α can be attributed to differing ISG induction at differing concentrations of IFN-α; low-dose IFN- α tends to induce anti-viral ISGs, whereas high-dose induces proliferation and inflammation-related ISGs (54). Our findings that low-dose IFN-α modulates breast cancer invasion and adhesion is notable in that it posits a potential anti-tumor clinical benefit through multiple mechanisms of action, without the morbidity of high-dose treatment.

Higher ISG expression is associated with estrogen receptor negative breast cancers (22). The cell lines we focused on in this study are both triple-negative breast cancers, and the RNAseq results of decreased ISG expression in RhoC knockout cells compared to wild-type were more significant in our triple-negative breast cancer cell lines than in other breast cancer cell lines (**Supplementary Table 1**). A recent study by Doherty et al. (55) also examined the effect of low-dose IFN-α on triple-negative breast cancer and found that chronic, weeks-long exposure to low-dose IFN-α led to increased epithelial morphology, decreased stemness markers, and decreased migration (55). This is consistent with our results of decreased invasion with 3 days of low-dose IFN-α treatment, and comparable to our results of both increased epithelial morphology and decreased invasion in RhoC knockout cells compared to wild-type. Previous work from our lab has identified RhoC as a modulator of stemness markers in breast cancer cells, and moreover identified RhoC as necessary for lung metastasis from orthotopic xenografts while increased stemness markers modulated the number of metastases (56). This study suggests that these previously discovered links to epithelial character, stemness and invasion in both IFN-α and RhoC may be, at least in part, related to RhoC’s contribution to IFN response.

Of note, we created crRhoA and crRhoC breast cancer cell lines and found that knocking out RhoC resulted in increased expression of RhoA while knocking out RhoA resulted in a smaller magnitude increase in RhoC expression (**Figure 1A**). Thus, our results in our crRhoC cells could be due to mixed effects from increases in RhoA as well as a lack of RhoC signaling. Practically speaking, however, a compensatory feedback loop of RhoA and RhoC is most likely active *in vivo*, so any therapeutic trials of RhoC inhibitors would need to also show benefit in the setting of increased RhoA expression. We also find that knocking out RhoC increased adhesion and junction stability to a similar extent in both MDA 231 and SUM 149 (**Figure 3**), but had differing effects in modifying IFN-α-driven inhibition of invasion in MDA 231 and SUM 149—transwell invasion was more inhibited by IFN-α treatment in crRhoC compared to wild-type SUM 149, while in MDA 231 IFN-α treatment inhibited transwell invasion to a greater extent in wild-type compared to crRhoC cells (**Figure 5B**). We propose that this differing effect on invasion but not on junction function may be due to a difference in STAT2 and pSTAT2 regulation, as we find that in SUM 149 IFN-α-treated cells RhoC knockout decreases STAT2 and pSTAT2 expression compared to wild-type while in MDA 231 IFN-α-treated cells RhoC knockout increases STAT2 and pSTAT2 expression compared to wild-type (**Figures 4B, C** **)**. Thus, cells with increased STAT2 and pSTAT2 upon stimulation with IFN-α were more resistant to IFN-α-driven transwell invasion inhibition, and furthermore RhoC expression affects STAT2 and pSTAT2 expression in different ways in inflammatory and non-inflammatory breast cancer cells. Further studies are needed to validate these findings in other inflammatory and non-inflammatory breast cancer cell lines and to assess the mechanism of action by which RhoC expression may modulate STAT2 expression.

Our overall hypothesis—that RhoC amplifies interferon signaling and thereby increases junction dysregulation, consequently promoting cancer cells’ motility and invasiveness—is borne out insofar as RhoC contributes to Type I interferon cellular response and also contributes to regulation of junction behavior. However, we find that IFN-α signaling itself results in increased cell-cell adhesion and decreased invasion. Our current work supports that the role of RhoC in metastases of certain aggressive cancers appears to be a result of intrinsic modulation of the cancer cells’ junctions and invasiveness, and potential amplification of interferon signaling; other effects on the tumor microenvironment, such as a shift in macrophage population abundance, may cooperate to produce highly aggressive phenotypes. As such, *via* multiple mechanisms, our data indicate that the inhibition of RhoC in aggressive breast cancers could provide anti-invasion therapeutic benefit.

## Data Availability Statement

The datasets presented in this study can be found in online repositories. The names of the repository/repositories and accession number(s) can be found below: https://www.ncbi.nlm.nih.gov/geo/, GSE175787.

## Author Contributions

HA: Performed experiments, analyzed data, wrote first draft of manuscript. PU: analyzed bioinformatics data, wrote bioinformatics methods, edited manuscript. LG: performed experiments JY: troubleshooted experiments, edited manuscript, assisted with figures. AL: performed experiments, analyzed data. LB: performed experiments. ZW: performed experiments. SM: suggested topic, funded work, mentored and directed experiments, edited manuscript. All authors contributed to the article and approved the submitted version.

## Funding

HA was partially supported by the University of Michigan Medical Scientist Training Program and the Sheth Research Fund. SDM was also supported by the Breast Cancer Research Foundation, the Ravitz Foundation, and the GreaterGood Charities.

## Conflict of Interest

The authors declare that the research was conducted in the absence of any commercial or financial relationships that could be construed as a potential conflict of interest.

## Publisher’s Note

All claims expressed in this article are solely those of the authors and do not necessarily represent those of their affiliated organizations, or those of the publisher, the editors and the reviewers. Any product that may be evaluated in this article, or claim that may be made by its manufacturer, is not guaranteed or endorsed by the publisher.
