# Supplementary material for: RhoC Modulates Cell Junctions and Type I Interferon Response in Aggressive Breast Cancers
Source: Front Oncol. 2021 Aug 26;11:712041. doi: 10.3389/fonc.2021.712041 (PMC8428533; doi:10.3389/fonc.2021.712041)
Supplement: Supplementary file 1 [file DataSheet_1.docx]

Supplementary Material

# Supplementary Figures

**
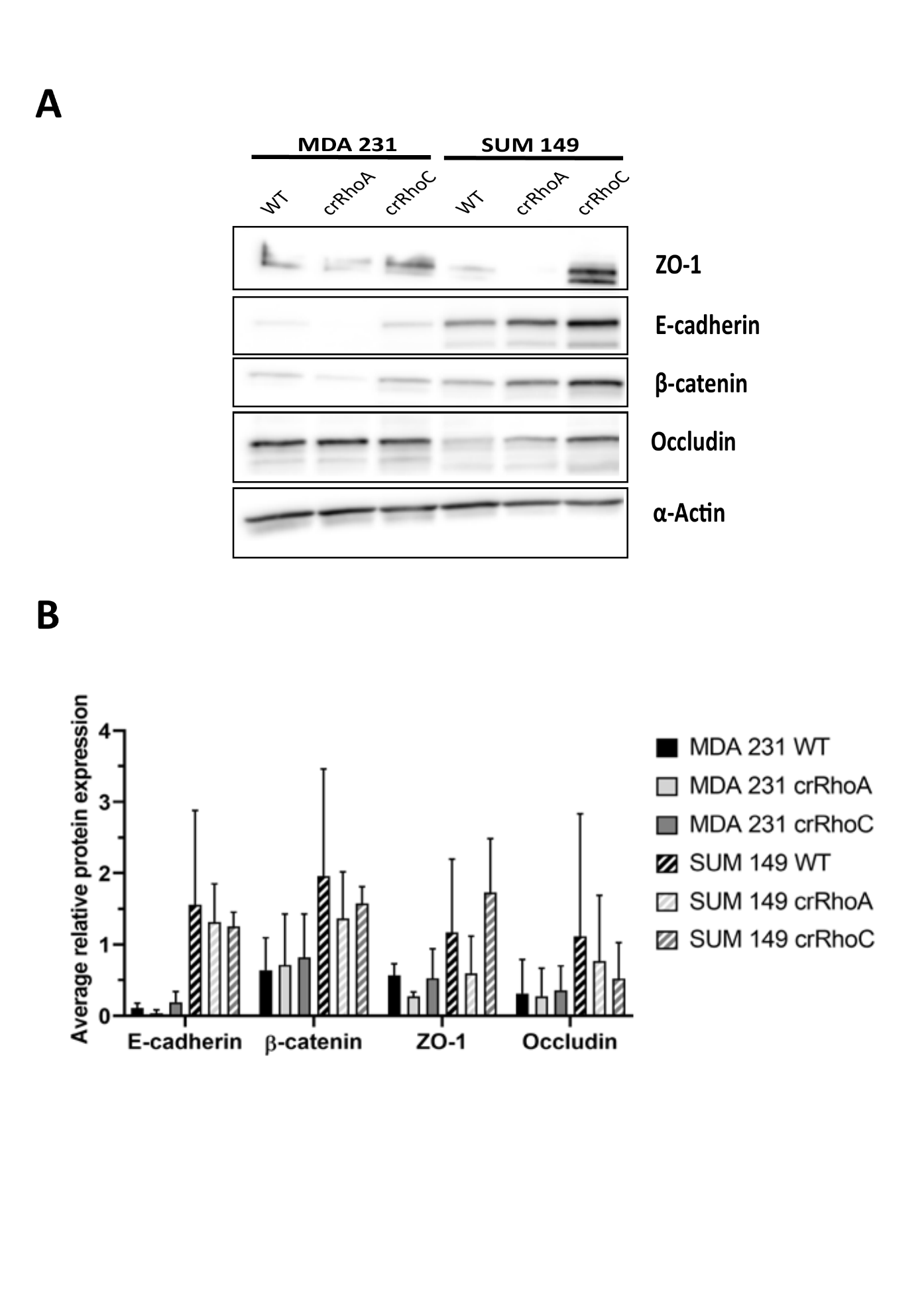
**

**Supplementary Figure 1: Rho knockout changes junction marker expression.** (A) Representative Western blot of junction protein expression in wild-type, crRhoA, and crRhoC cells. crRhoC cells trended towards increased expression of junction markers, most notably ZO-1. (B) Quantification of Western blots of three biological replicates. No changes in junction expression reached significance. Solid bars are MDA 231, striped bars are SUM 149; black bars are wild-type, light grey bars are crRhoA cells, and dark grey bars are crRhoC cells.

**
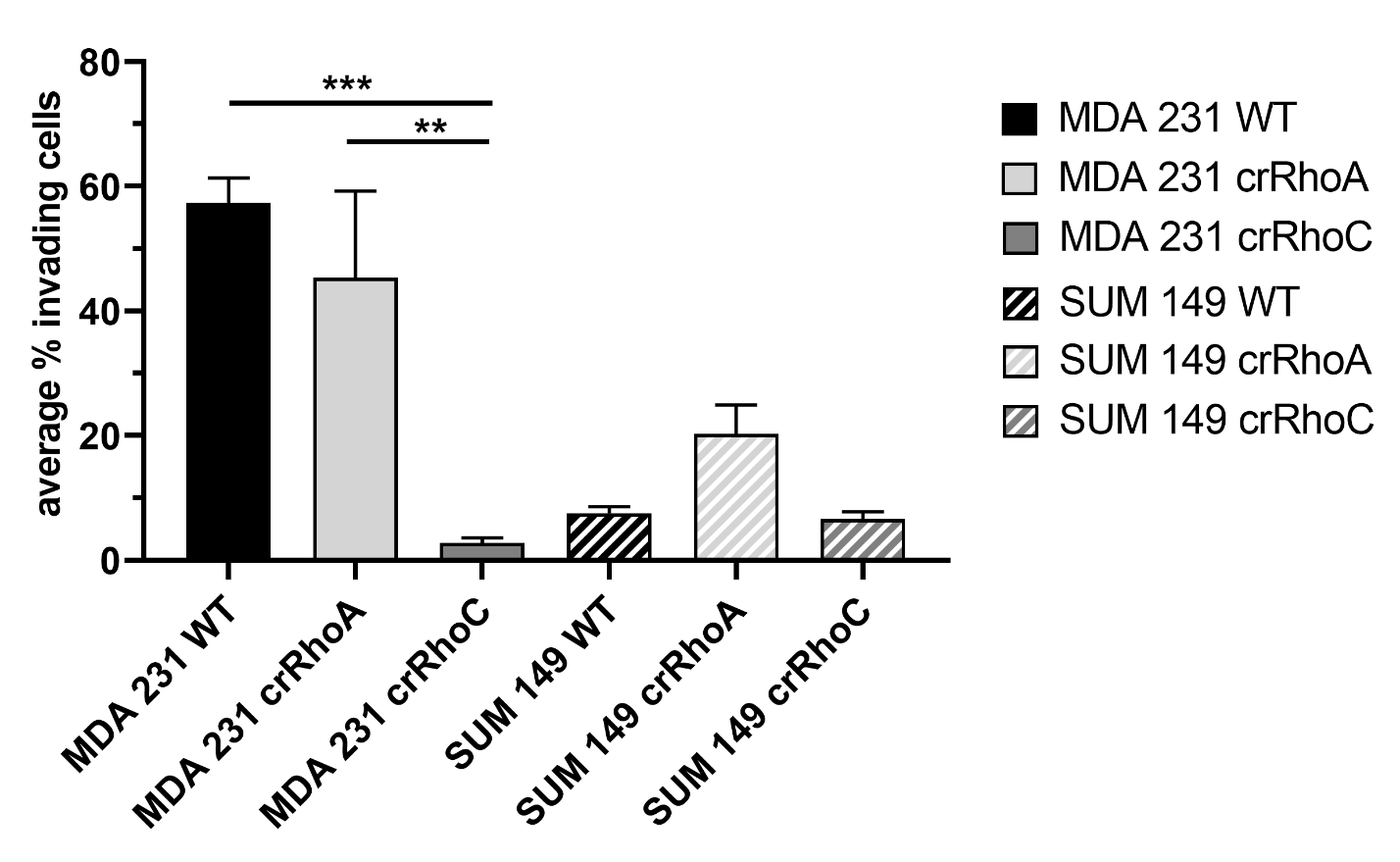
**

**Supplementary Figure 2: Rho knockout changes breast cancer cell invasiveness.** In Matrigel-coated Transwell invasion assays (n = 3 biological replicates), crRhoC cells were less invasive than wild-type cells, most significantly in MDA 231. Solid bars are MDA 231, striped bars are SUM 149; black bars are wild-type, light grey bars are crRhoA cells, and dark grey bars are crRhoC cells. **: p < 0.01; ***: p < 0.001.


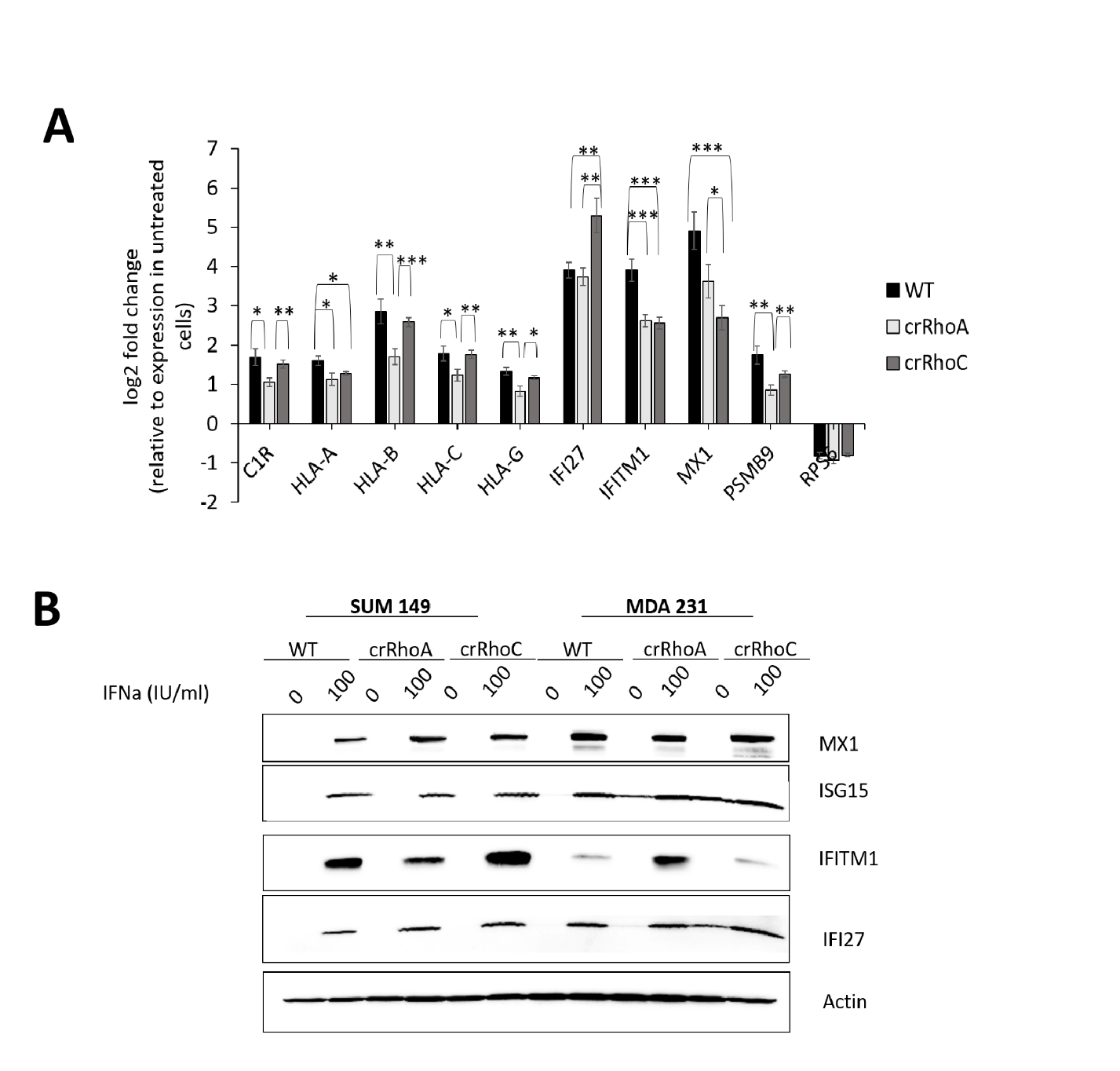


**Supplementary Figure 3: Rho knockout changes protein expression of interferon-stimulated genes. (A)** RNA expression of interferon stimulated genes in response to 72 hours of IFN-α treatment (100 IU/ml) in MDA 231 and SUM 149 cells. Wild-type cells had larger increases in gene expression with IFN-α treatment compared to crRhoA and crRhoC cells. Black bars are expression in wild-type cells, light grey bars are expression in crRhoA cells, dark grey bars are expression in crRhoC cells. *: p < 0.05; **: p < 0.01; ***: p < 0.001. **(B)** Protein expression of interferon stimulated genes in response to 72 hours of IFN-α treatment (100 IU/ml).

# Supplementary Tables

**Supp. Table 1: IRF9 and STAT2 are predicted inhibitory regulators in crRhoC cells compared to wild-type.** iPathwayGuide analysis of RNAseq data identified multiple genes downregulated in crRhoC compared to wild-type cells (“consistent (-)”) that were also identified as downstream of IRF9 and STAT2 based on KEGG (“DE targets”). Significance determined by p < 0.05 and indicated by *.

|  | **crRhoC vs WT** | | | | | | | | | |
| --- | --- | --- | --- | --- | --- | --- | --- | --- | --- | --- |
|  | **SUM 149** | | **MDA 231** | | **VARI 068** | | **SUM 190** | | **MCF7** | |
| **Gene** | Consistent (-)/DE targets | FDR-adjusted p-value | Consistent (-)/DE targets | FDR-adjusted p-value | Consistent (-)/DE targets | FDR-adjusted p-value | Consistent (-)/DE targets | FDR-adjusted p-value | Consistent (-)/DE targets | FDR-adjusted p-value |
| IRF9 | 13/15 | 0.148 | 15/16* | 0.003 | 23/23* | 2.29e-14 | 0/1 | 1.00 | 5/5 | 0.736 |
| STAT2 | 13/15 | 0.230 | 15/17* | 0.018 | 24/24* | 2.29e-14 | 0/1 | 1.00 | 5/5 | 0.736 |

**Supp. Table 2: Interferon-stimulated genes are downregulated in crRhoC cells compared to wild-type.** Combining the RNAseq results of crRhoC vs WT in all cell lines assessed (SUM 149, MDA 231, VARI 068, SUM 190, and MCF7), 17 genes downstream of IRF9 and STAT2 were significantly downregulated in crRhoC cells compared to wild-type, with significance determined by p < 0.05.

| **Gene** | **LogFC** | **FDR-adjusted p-value** |
| --- | --- | --- |
| IFI27 | -2.90 | 1.00e-6 |
| BST2 | -2.10 | 1.00e-6 |
| RSAD2 | -1.81 | 1.00e-6 |
| IFI6 | -1.31 | 1.00e-6 |
| OAS2 | -1.27 | 2.29e-4 |
| XAF1 | -1.20 | 1.00e-6 |
| MX1 | -1.07 | 1.00e-6 |
| PSMB8 | -1.00 | 1.00e-6 |
| IFIT1 | -0.886 | 3.59e-4 |
| IFIT3 | -0.783 | 3.133-4 |
| IFI35 | -0.758 | 1.00e-6 |
| ISG20 | -0.756 | 2.03e-4 |
| IFITM3 | -0.681 | 1.00e-6 |
| OASL | -0.678 | 0.002 |
| IFIT2 | -0.673 | 0.004 |
| OAS3 | -0.667 | 3.01e-4 |
| IRF5 | -0.610 | 1.36e-5 |
